# Discrete subpulmonic membrane in association with isolated severe pulmonary valvar stenosis

**DOI:** 10.1186/1471-2261-13-43

**Published:** 2013-06-21

**Authors:** Endale Tefera, Ramón Bermudez-Cañete, Lola Rubio

**Affiliations:** 1 Department of Pediatrics & Child Health, School of Medicine, Addis Ababa, University & Cardiac Center, Addis Ababa, Ethiopia; 2 Department of Pediatric Cardiology, Cardiac Center Ethiopia, Ramon y Cajal University Hospital, Madrid, Spain; 3 Department of Pediatric Cardiology, Cardiac Center Ethiopia, La Paz Hospital, Madrid, Spain

**Keywords:** Subpulmonic membrane, Right ventricular outflow, Pulmonary stenosis, Percutaneous valvotomy

## Abstract

**Background:**

Subpulmonic membrane as a cause of right ventricular outflow tract obstruction in patients with concordant ventriculoarterial connection and intact ventricular septum is considered to be rare.

**Case presentation:**

A 7 – year – old boy was referred to a tertiary care hospital with complaints of dyspnea on moderate exertion and palpitations of about 2 years duration. Physical examination revealed parasternal lift, systolic thrill and a 4/6 ejection systolic murmur, best heard over the left 2^nd^ intercostal space. His oxygen saturation was 88% on room air. Two-dimensional echocardiography showed a thickened pulmonary valve with fused leaflets that show severe systolic doming. There was a discrete subpulmonic membrane about 1.3 cm below the pulmonary valve annulus. Continuous wave Doppler interrogation showed peak systolic pressure gradient of 185 mmHg across the pulmonary valve. Balloon dilation of the pulmonary valve was performed and the pressure gradient came down to 50 mmHg. Follow-up transthoracic echocardiography showed residual pressure gradient of about 50 – 60 mmHg across the pulmonary valve. The residual pressure gradient appeared to be mainly subvalvar, as seen on the continuous wave Doppler tracing. The patient reported marked improvement in terms of exercise tolerance and subjective symptoms.

**Conclusions:**

Association of subpulmonic membrane with severe pulmonary valvar stenosis, concordant ventriculoarterial connection and intact ventricular septum is rare. When it occurs, the result of percutaneous valve dilation may be suboptimal.

## Background

Membranous subpulmonic stenosis in association with supracristal ventricular septal defect or with ventricular septal defect and aortic regurgitation has been reported in recent literature [1,2]. Subpulmonic membrane in patients with corrected transposition of the great arteries has also been reported and in this setting it has also been mentioned that percutaneous balloon dilation of the membranous obstruction was effective [3]. Isolated subpulmonic membrane causing critical neonatal subpulmonic stenosis has also been described [4]. However, subpulmonic membrane in association with isolated pulmonary valvular stenosis is not well described at least to our knowledge.

## Case presentation

We report a 7 year old boy who was referred to the Cardiac Center in Addis Ababa with dyspnea on moderate exertion and palpitations of about 2 years duration. The child was found to have a cardiac murmur at the age of about 2 years but was not subjected to echocardiography due to lack of skill and equipment in his locality. He was not on any medication at the time of presentation.

On physical examination, he appeared to be well-grown and healthy-appearing boy with a weight of 39 kg, height of 132 cm and body mass index of 22.4. His blood pressure was 127/55 mmHg and his pulse rate was 84 bpm. His oxygen saturation was 88% on room air. There was no remarkable cyanosis or clubbing of the fingers. Peripheral pulses were palpable. He had a parasternal lift, systolic thrill and a 4/6 ejection systolic murmur, best heard over the left 2^nd^ intercostal space and a pansystolic murmur of grade 3/6 intensity, best heard over the left lower sternal border. There was no hepatomegaly or peripheral edema.

**Figure 1:**
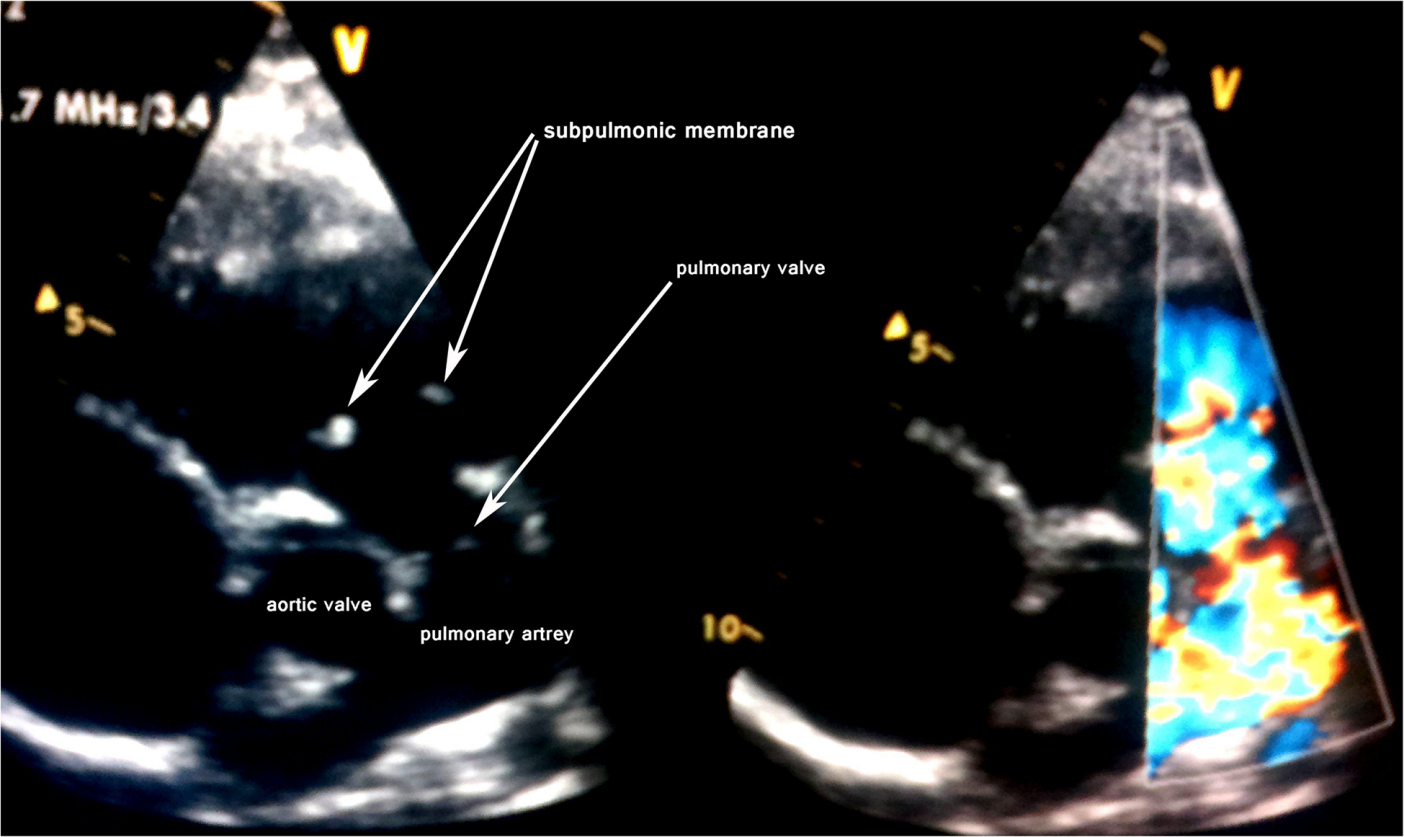
Two-dimensional and color flow echocardiographic frames showing discrete membrane below the pulmonary valve (post balloon dilation of the pulmonary valve).

His chest X – ray showed marked cardiac enlargement with clearly decreased pulmonary vascular markings. The electrocardiogram showed sinus rhythm with a rate of 88 beats per minute, incomplete right bundle branch block, right ventricular hypertrophy, right atrial enlargement and right axis deviation. Echocardiographic examination showed normal abdominal and atrial situs, with normal position of the heart in the left chest and normal venoatrial, atrioventricular and ventriculoarterial relationships. The right ventricle looked markedly dilated and hypertrophied. The right atrium looked dilated. Right ventricular systolic function appeared grossly reduced on visual inspection and Tricuspid Annular Plane Systolic Excursion (TAPSE). There was a patent foramen ovale with right to left flow by color Doppler. The interventricular septum appeared intact. The mitral and aortic valves were structurally normal. The tricuspid valve looked thickened. The pulmonary valve was thickened with fused leaflets. No calcification was seen. There was a trickle of flow seen across the valve. There was a discrete circumferential membrane about 1.3 cm below the pulmonic valve (Figure 1). Continuous wave Doppler across the pulmonic valve showed a peak systolic pressure gradient of 185 mmHg. However, pulsed wave Doppler just distal to the membrane did not show significant turbulence or pressure gradient before dilation of the valve. He had also moderate tricuspid regurgitation with pressure gradient of 185 mmHg. No pericardial effusion was seen.

**Figure 2:**
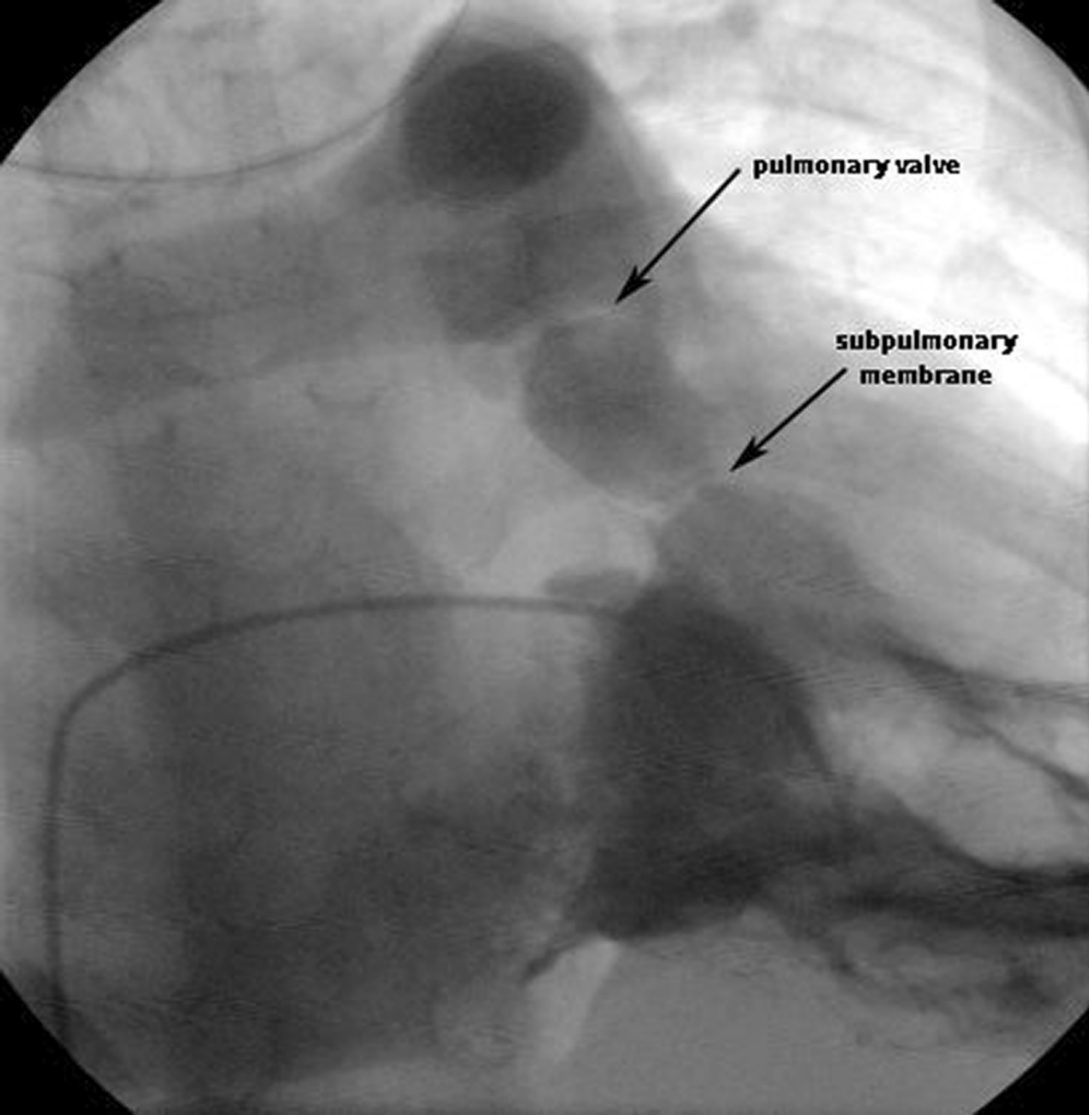
RV angiogram in the frontal projection with cranial angulation, showing discrete membrane below the pulmonary valve.

**Figure 3:**
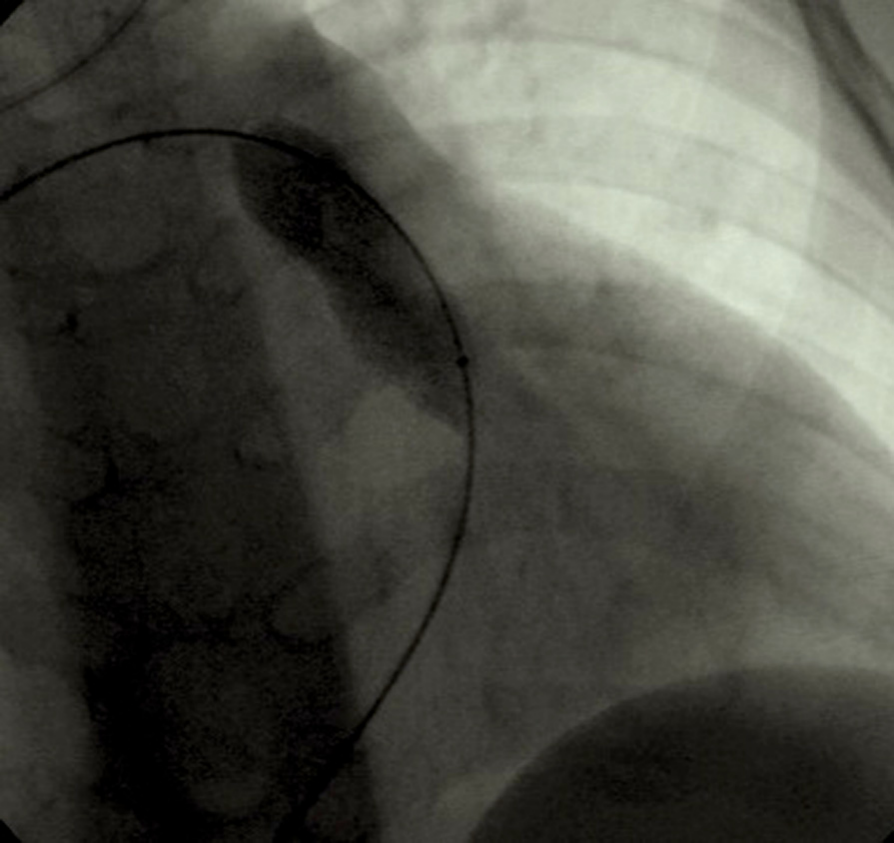
17 mm × 30 mm dilation Balloon inflated across the pulmonary valve (frontal projection with cranial angulation).

The boy was scheduled for percutaneous balloon dilation of the valve under general anesthesia and was taken to the catheterization laboratory. Right ventriculography in the frontal projection showed a discrete membranous obstruction about 1.3 cm below the pulmonic valve (Figure 2). Right ventricular systolic pressure was 160 mmHg and the pulmonary arterial systolic pressure was 15 mmHg. With a 17 mm × 30 mm Osypka balloon, dilation of the valve was performed (Figure 3). Post-dilation right ventricular pressure was 72 mmHg and that of the pulmonary artery was 22 mmHg. There was mild post-dilation pulmonary regurgitation. After dilation of the valve, both catheter measurement and echocardiography showed significant subvalvar pressure gradient. The patient tolerated the procedure and there was no complication. He was extubated in the catheterization laboratory. He was discharged after one day in hospital. He was given propranolol 10 mg orally 3 times a day for 3 months. His discharge echo showed reduction in the degree of tricuspid regurgitation, mild pulmonic regurgitation and a subvalvar right ventricular outflow tract gradient of 45-50 mmHg. On a 1-month follow up visit, he reported significant improvement in his clinical symptoms and improved exercise capacity. His oxygen saturation on this visit was 95% at room air. His right ventricular outflow gradient on this visit *was* approximately 60 mmHg and his tricuspid regurgitation has decreased in severity. His right ventricular function has improved.

## Discussion

Subpulmonic obstructions are usually due to infundibular hypertrophy or subpulmonic muscle bundles. Other causes of subpulmonic obstruction reported in the literature include aneurysm of the membranous septum [5,6], aneurysmal tricuspid valve tissue in the setting of membranous ventricular septal defect [7], double-chambered right ventricle [8], and unruptured sinus of Valsalva aneurysm [9,10]. A single case of right ventricular outflow tract obstruction resulting from a tuberculoma in a patient with ventricular septal defect and aneurysm of the membranous septum has also been reported [11]. Subpulmonic membrane as a cause of right ventricular outflow tract obstruction is a rare occurrence [1]. Subpulmonic membrane in association with a supracristal ventricular septal defect has recently been reported by Duggal and coworkers [1]. Raff et al. also described a case of membranous subpulmonic stenosis associated with ventricular septal defect and aortic insufficiency where the membrane was incidentally noted on cardiac catheterization [2].

Our patient had severe pulmonary valvar stenosis with suprasystemic right ventricular pressure. With balloon dilation of the valve it was possible to lower the peak systolic pressure gradient from 185 mmHg down to a maximum of 50 mmHg. His post dilation echo suggested that the residual gradient was mainly due to subvalvar component. Though there is a report of success in dilating the membrane itself [3], it is understandable that balloon dilation of the valve is less likely to result in complete elimination of the right ventricular outflow tract gradient. Surgery is the currently preferred modality of treatment, which can result in complete resolution of the right ventricular out flow tract obstruction [4]. The interatrial shunt could be closed at the same time. When the subpulmonic membrane is too close to the pulmonary valve one needs to be cautious, as there is a risk of damage to the valve leaflets during surgery [4]. In our patient, the surgical option may be considered in the future based on the trend of the pressure gradient on subsequent follow up visits.

## Conclusion

Subpulmonic membrane as an isolated cause of right ventricular outflow tract obstruction is rare. It’s association with isolated valvar pulmonary stenosis and intact ventricular septum is even less common. When such association occurs, the result of percutaneous valve dilatation may be suboptimal. Unlike the usual case of infundibular hypertrophy with valvular stenosis, membranous stenosis may not be expected to respond to beta-blocker therapy after balloon valvoplasty and such patients may need surgical intervention from the outset or after attempted balloon dilation.

## Consent

Written informed consent for participation in this case study was obtained from parents of the child.

## Competing interests

The authors declare that they have no competing interests.

## Authors’ contributions

ET: conceived the case study and design and worked on the draft manuscript. RBC: revised the manuscript critically and contributed to the content. LR: revised the manuscript critically. All authors have read and approved the final version of the manuscript.

## Authors’ information

ET: is a pediatric cardiologist working at the cardiac catheterization laboratory of the cardiac center Ethiopia (a charity center) within the premises of school of medicine of Addis Ababa University.

RBC: is an interventional cardiologist coming as a volunteer to the cardiac center, Ethiopia.

LR: is a pediatric cardiologist coming as a volunteer to cardiac center, Ethiopia.

## Pre-publication history

The pre-publication history for this paper can be accessed here:

http://www.biomedcentral.com/1471-2261/13/43/prepub
